# 

**Published:** 1818-04

**Authors:** 


### CATALOGUE OF MEDICAL BOOKS.

Observations on Phagedæna Gangrænosa, in two Parts. By H. Home Blackadder, Surgeon. 8vo. 6s.

Observations on the Cure and Prevention of the Contagious Fever now prevalent in the City of Edinburgh and its Environs; by J. Yule, M.D. &c. 8vo. 2s. 6d.

The Morbid Anatomy of some of the most important Parts of the Human Body; by Matthew Baillie, M.D. Fifth edition, corrected. 8vo. 10s. 6d.

A Treatise on Blood-letting in Fever, by J. Van Rotterdam, Chemical Professor of Therapeutics and Materia Medica at Ghent, &c. Translated from the French by J. Taylor, M.D. 8vo. 10s.

Observations on several Diseases of the Eye, and Remarks on the Introduction of the Male Catheter, and on the Treatment of Hæmorrhoids; by the late James Ware, F.R.S. Edited by his Son, Martin Ware, Member of the Royal College of Surgeons. 8vo. 8s.

Surgical Essays; by Astley Cooper, F.R.S. &c. and Benjamin Travers, F.R.S. &c. Part I. 8vo. boards, 10s. 6d.

A Practical Introduction to Botany; illustrated by References, under each Definition, to Plants of easy access, and by numerous Figures; and also comprising a Glossary of Botanic Terms. By the Rev. W. Bingley, A.M. F.L.S. 12mo. 3s. 6d.

The Naturalist's Pocket-Book, or Tourist's Companion. By George Graves, F.L.S. 8vo. coloured, 1l. 1s. uncoloured, 14s.

Report of the House of Recovery and Fever Hospital of the City of Cork, from November 8th, 1816, to November 8th, 1817; containing Observations on the occasional Causes and Prevention of the present Epidemic Fever. By John Milner Barry, M.D. 8vo. 2s.

Observations relating to the Treatment, by Sir William Adams, of the Ophthalmic Cases of the Army. By John Vetch, M.D. Physician to the Forces. 8vo. 1s. 6d.

A Treatise on Sea-Bathing; with Remarks on the Use of the Warm Bath. By A. P. Buchan, M.D. Third Edition, with Additions. 8vo. boards, 5s.

---

### *NOTICES TO CORRESPONDENTS.*

*Our Leicester Correspondent seems very little acquainted with this large town. He may depend on it, the whole is a trick. If he wishes to answer either the Morning Post or Mr. ——, or to notice a work little known, he will do all the editors of the latter wish for, and all they mean by their feeble attempts. It must, however, not be through the medium of our Journal. We have perpetual assaults of the same kind, the only object of which we consider an attempt at notoriety, by gaining a corner among our valued correspondents. We advise Dr. P. to reflect with us,* "Multo majoris alapæ mecum veneunt." *Dr. P.'s letter shall be returned in any manner he will direct us.*

*The following favours, among other communications, shall appear in our next*—Dr. Kinglake's, Mr. Littleton, Mr. Syer. *Others in their turn.*

